# Alkohol und Trauma: Retrospektive Analyse von traumatologischen Schockraumpatienten auf Alkoholkonsum an einem universitären Maximalversorger

**DOI:** 10.1007/s00101-025-01546-1

**Published:** 2025-06-18

**Authors:** Josina Straub, Julia Elisabeth Lenz, Markus Zimmermann, Manuela Malsy

**Affiliations:** 1 Klinik und Poliklinik für Unfallchirurgie, Universitätsklinikum Regensburg, Franz-Josef-Strauss Allee 11, 93053 Regensburg, Deutschland; 2 Abteilung für klinische Akut- und Notfallmedizin, Universitätsklinikum Regensburg, Regensburg, Deutschland; 3 Klinik für Anästhesiologie, Universitätsklinikum Regensburg, Regensburg, Deutschland

**Keywords:** Unfallmechanismus, Traumatologischer Schockraum, Alkoholintoxikation, Injury Severity Score, Trauma mechanismen, Resuscitation room, Alcohol intoxication, Injury severity score

## Abstract

**Hintergrund:**

Seit Langem ist bekannt, dass es einen kausalen Zusammenhang zwischen dem Alkoholkonsum einer Bevölkerung und der Häufigkeit von Unfällen gibt. Seit 1975 werden durch das Statistische Bundesamt Daten über Verkehrsunfälle mit polizeilich registriertem Personenschaden erhoben. Bei der Bewertung der Daten ist von einer erheblichen Dunkelziffer auszugehen, da nicht bei jedem Unfallbeteiligten konsekutiv der Ethanolgehalt im Blut erhoben wird. Die vermutlich beste Möglichkeit, alkoholbedingte Unfälle und Verletzungen zu erforschen, bieten daher Studien aus Notaufnahmen. Ziel der vorliegenden Studie war es, Patienten, die als traumatologischer Schockraum in einem universitären Maximalversorger vorstellig wurden, hinsichtlich einer konsekutiven Alkoholintoxikation zu untersuchen.

**Methodik:**

Es erfolgte die retrospektive Analyse aller traumatologischen Schockraumpatienten eines Maximalversorgers in Bayern ab dem 14. Lebensjahr, die von Anfang Januar 2021 bis Ende Dezember 2024 vorstellig wurden. Hierbei wurden neben dem Ethanolgehalt im Blut der Unfallmechanismus, die Schwere der Verletzung anhand des Injury Severity Score (ISS) sowie der tödliche Verlauf erfasst.

**Ergebnisse:**

Insgesamt wurden 1780 verunfallte Patienten eingeschlossen. Die häufigsten Unfälle waren Verkehrsunfälle (58,7 %), gefolgt von Treppenstürzen (8,3 %) und Stürzen aus großer Höhe (8,2 %). Insgesamt konnte bei 16,1 % aller eingelieferten Patienten Ethanol im Blut nachgewiesen werden. Die Gammaglutamyltransferase(γ-GT)-Aktivität zeigte sich im Durchschnitt mit 75,1 U/l bei alkoholisierten Patienten erhöht. Zeitlich wurden in den späten Abend- und frühen Morgenstunden sowie am Wochenende die meisten alkoholintoxikierten Patienten beobachtet. Uhrzeitlich verunfallten die eingelieferten Personen am häufigsten zwischen 12 und 20 Uhr, wobei etwa jeder 10. Patient einen Blutalkoholspiegel ≥ 0,2 ‰ aufwies. In der Zeit zwischen 0 Uhr und 4 Uhr konnte bei 43,8 % der eingelieferten Schockraumpatienten Ethanol im Blut nachgewiesen werden. Der durchschnittliche Blutalkoholspiegel lag bei 1,55 ‰.

**Diskussion:**

Alkohol ist auch heute noch ein entscheidender Risikofaktor für Unfälle, Verletzungen und Sterblichkeit. Die vorliegende Studie liefert wichtige Daten zum wahren Ausmaß alkoholbedingter Unfälle in der Schwerverletztenversorgung. Resultierend muss das Bewusstsein für den Risikofaktor Alkohol geschärft und wieder in den Fokus gestellt werden. Denn nur durch zusätzliche volksnahe, präventive Maßnahmen und ggf. Verbote kann eine Abnahme risikoreichen Alkoholkonsums in der Bevölkerung erreicht werden.

## Abstract (en)

**Background:**

A well-established causal relationship exists between alcohol consumption in a population and the frequency of accidents. Since 1975 the German Federal Statistical Office has systematically recorded data on traffic accidents involving personal injuries as reported by the police: however, these data are likely to underestimate the true burden due to incomplete testing of blood ethanol levels among the individuals involved. Emergency department-based studies provide a more accurate framework for investigating alcohol-related accidents and injuries. This study aimed to assess the prevalence of alcohol intoxication among consecutive patients admitted to the trauma room of a tertiary care university hospital.

**Methods:**

A retrospective analysis was conducted, including all patients aged 14 years and older admitted to the trauma room of a level I trauma center in Bavaria between January 2021 and December 2024. Parameters assessed included blood ethanol concentrations, mechanisms of injury, injury severity quantified by the injury severity score (ISS) and fatal outcomes.

**Results:**

The study cohort comprised 1780 trauma patients. The leading mechanism of injury was traffic accidents (58.7%), followed by stair-related falls (8.3%) and falls from a height (8.2%). Ethanol was detected in the blood in 16.1% of patients, with an average gamma-glutamyltransferase (γGT) activity of 75.1 U/l in alcohol intoxicated patients. Temporal patterns revealed a higher incidence of alcohol intoxication during late evening and early morning hours and on weekends. Accidents were most frequently reported between 12:00h and 20:00h, with 10% of patients presenting with a blood alcohol concentration exceeding ≥ 0.2 ‰. Notably, 43.8% of patients admitted between 00:00h and 04:00h tested positive for ethanol, with a mean blood alcohol concentration of 1.55 ‰.

**Discussion:**

Alcohol consumption is still a decisive risk factor for accidents, injuries and mortality. This study provides a comprehensive analysis of the role of alcohol in severe trauma cases, emphasizing the urgent need for targeted public health strategies. Enhanced preventive measures, public education and stricter regulations are critical to mitigate the risks associated with alcohol-related accidents and reduce the societal impact.

International ist Alkohol einer der führenden Risikofaktoren für Unfälle und Verletzungen. Trotz eines erheblichen Rückgangs schwerer Verkehrsunfälle in den letzten Jahren ist Alkohol nach wie vor ein Hauptrisikofaktor für vorzeitige Sterblichkeit. Im Jahr 2021 starben etwa 1,8 Mio. Menschen weltweit an den Folgen ihres Alkoholkonsums. In Deutschland konsumieren 9 Millionen Menschen Alkohol in problematischer Weise und 1,6 Millionen gelten als alkoholabhängig [6]. Alkoholbedingte Verletzungen sind nicht nur ein Problem einer kleinen, stark konsumierenden Minderheit, sondern betreffen große Bevölkerungsteile. In dieser Arbeit wird das Ausmaß der konsekutiven Alkoholintoxikation bei traumatologischen Schockraumpatienten eines Maximalversorgers in Bayern von Januar 2021 bis Dezember 2024 analysiert.

Bereits seit Langem ist bekannt, dass es einen kausalen Zusammenhang zwischen dem Alkoholkonsum einer Bevölkerung und der Häufigkeit von Unfällen oder Verletzungen gibt [1]. So können viele, akute Verletzungen in der Notaufnahme auf Alkoholkonsum zurückgeführt werden [2]. In zahlreichen Studien wurde dieser kausale Zusammenhang auch in Laborstudien bestätigt. Durch Alkoholkonsum kommt es durch eine akute Vergiftung des Körpers zu Koordinations‑, Sprach- und Sehstörungen sowie zu Verwirrtheit und Orientierungsstörungen [3]. Alkohol gilt zudem als intaktogen, also als eine Substanz „die Gemeinschaft stiftet“, und ist daher zu einem zentralen Bestandteil bei vielen Festen und Feierlichkeiten geworden [4]. Nach Angabe des globalen Statusberichts der Weltgesundheitsorganisation gehörte Deutschland im Jahr 2019 zu den Ländern mit dem weltweit höchsten Pro-Kopf-Alkoholkonsum [5]. So trinkt jeder Erwachsene im Schnitt pro Jahr etwa 12,2 l reinen Alkohol, entsprechend ca. 240 l Bier [6], und 9 Mio. Menschen haben einen problematischen Alkoholkonsum. Mit dem Begriff „problematischer Alkoholkonsum“ werden dabei Verhaltensweisen beschrieben, bei denen der Alkoholkonsum zu negativen Folgen, wie körperlichen oder psychischen Beeinträchtigungen, sozialen oder beruflichen Problemen oder zu Kontrollverlust, geführt haben. In Deutschland gelten 1,6 Mio. Menschen als alkoholabhängig [7].

Seit 1975 werden durch das Statistische Bundesamt Daten über Verkehrsunfälle mit polizeilich registriertem Personenschaden unter dem Einfluss berauschender Mittel erhoben. Dabei zeigt sich, dass sich insgesamt die Zahl der Alkoholunfälle mit Personenschaden seit 1975 um 73,6 % von 51.593 auf 13.628 im Jahr 2021 verringerte. Ferner verunglückte im Jahr 2021 bei Alkoholunfällen nur noch gut ein Fünftel der Personen von 1975, nämlich 16.426 anstelle von 76,578. Starben 1975 noch 21,4 % aller Verkehrstoten an den Folgen eines Alkoholunfalls, so waren es 2021 nur noch 6,4 %. Im Jahr 2021 wurde jedoch wieder ein Anstieg alkoholbedingter Unfälle im Vergleich zum Vorjahr (+4,8 %) beobachtet [8].

Bei der Bewertung der Daten ist von einer erheblichen Dunkelziffer auszugehen, da nicht bei jedem Unfallbeteiligten festgestellt wird, ob dieser unter dem Einfluss von Alkohol gestanden hat. Zudem werden Alleinunfälle, wie Leiter- oder Treppenstürze, aber auch Verkehrsunfälle bei dem, außer dem Fahrer, niemand weiteres beteiligt war, häufig polizeilich nicht registriert [8].

Die vermutlich beste Möglichkeit, alkoholbedingte Unfälle und Verletzungen zu erforschen, bieten daher Studien aus Notaufnahmen. Doch aktuelle Arbeiten hierzu gibt es kaum. Eine Studie aus der Notaufnahme der Lausanner Universitätsklinik aus dem Jahr 1993 von Yersin et al. zeigte, dass etwa 21 % aller Verkehrsunfälle, 5 % aller Arbeitsunfälle, 17 % aller Haushaltsunfälle und 35 % aller Unfälle im öffentlichen Raum als alkoholbedingt ermittelt wurden. Alkoholbedingt hieß in diesem Rahmen eine Blutalkoholkonzentration von 0,8 ‰ oder mehr. Eine weitere Studie aus dem Universitätsspital Lausanne aus dem Jahr 2007 zeigte, dass knapp 17 % aller Verletzungen bei Männern und 12 % bei Frauen auf Alkoholkonsum zurückzuführen waren [9].

Ziel der vorliegenden Arbeit war es, alle Patienten, die als traumatologischer Schockraum in einem universitären Maximalversorger vorstellig wurden, hinsichtlich einer konsekutiven Alkoholintoxikation zu untersuchen.

## Methodik

### Datenerfassung

Es erfolgte die retrospektive Analyse aller Schockraumpatienten eines Maximalversorgers in Bayern. Hierfür wurden alle Patienten, welche über den traumatologischen Schockraum im Universitätsklinikum Regensburg von Anfang Januar 2021 bis Ende Dezember 2024 vorstellig wurden, analysiert. Eingeschlossen wurden alle Patienten ab dem 14. Lebensjahr, da diese laut dem Jugendschutzgesetz in Deutschland im Beisein eines Erziehungsberechtigten Alkohol konsumieren dürfen. Zeitlich wurden alle schockraumgeführten Notfälle, 24 h die Woche, registriert. Hierbei wurden neben dem Ethanolgehalt im Blut der Unfallmechanismus, die Schwere der Verletzung anhand des Injury Severity Score (ISS) sowie der tödliche Verlauf erfasst. Es erfolgte die Bestimmung der Laborwerte, inklusive Ethanolspiegel, entsprechend einer geltenden, hausinternen SOP für traumatologische Schockraumpatienten. Da nach den Erkenntnissen der internationalen Flugmedizin bereits bei 0,2 ‰ eine Leistungsbeeinträchtigung vorliegt, wurden Patienten ab einem Ethanolspiegel von 0,2 ‰ als alkoholisiert gewertet.

Des Weiteren wurden die laborchemischen Parameter, wie das mittlere, korpuskuläre Volumen (MCV) der Erythrozyten und die Gammaglutamyltransferase(γ-GT)-Aktivität bei alkoholisierten und nichtalkoholisierten Patienten betrachtet, um auf einen möglichen, chronischen Alkoholkonsum der Patienten schließen zu können [10]. Die laborchemische Untersuchung erfolgte unmittelbar nach Einlieferung des Patienten. Insgesamt konnten 1780 Patientendaten analysiert werden.

### Statistische Analyse

Die Studie wurde von der Ethikkommission der Universität Regensburg (ID: 24-3999-104) genehmigt und gemäß der Deklaration von Helsinki durchgeführt. Die Daten wurden untersucht, um die Prävalenz einer konsekutiven Alkoholintoxikation bei Patienten, die in einem traumatologischen Schockraum in einem universitären Maximalversorger vorstellig wurden, zu identifizieren. Die statistische Analyse wurde mit dem Statistikprogramm SPSS (IBM SPSS Statistics, Version 30.0.0, Zürich, Schweiz) durchgeführt. Kategoriale Variablen wurden in Form von Häufigkeiten und Prozentangaben dargestellt. Deskriptive Statistiken wurden berechnet, um die Merkmale der traumatologischen Schockraumpatienten aufzuzeigen. Kontinuierliche Variablen wurden als Mittelwert und Standardabweichung präsentiert, kategoriale Variablen als Anzahl der Beobachtungen und Häufigkeiten.

## Ergebnisse

Von Anfang Januar 2021 bis Ende Dezember 2024 wurden insgesamt 1780 verunfallte Patienten als traumatologischer Schockraum im Universitätsklinikum Regensburg vorstellig. 31 % davon waren Frauen und 69 % der Verletzten Männer. Die häufigsten Unfälle waren Verkehrsunfälle (58,7 %), gefolgt von Treppenstürzen (8,3 %) und Stürzen aus großer Höhe (8,2 %). Dabei wurden insgesamt 347 berufsgenossenschaftliche Unfälle registriert. Das durchschnittliche Alter betrug 46,1 Jahre (Tab. 1).

**Tab. 1:** Epidemiologische Daten aller traumatologischer Schockraumpatienten der Jahre 2021–2024

	Gesamtanzahl	2021	2022	2023	2024
*Traumatologische Schockraumpatienten*					
Anzahl	1780	495	488	383	414
Weiblich	552	173	157	105	117
Männlich	1228	322	331	278	297
*Unfallarten*					
Verkehrsunfälle	1044	312	290	208	234
Treppenstürze	148	43	40	37	28
Sturz aus großer Höhe	146	29	45	19	53
Stolpersturz	138	31	39	35	33
Leiterstürze	89	36	17	25	11
Einklemmung	67	7	14	25	21
Messerstecherei	41	8	10	11	12
Reitunfall	32	13	9	3	7
Forstunfall	23	3	3	9	8
Schlägerei	20	3	5	6	6
Andere	32	10	16	5	1
*Anteil berufsgenossenschaftlicher Unfälle*	19,6 %	18,4 %	17,6 %	21,4 %	21,7 %
*Durchschnittsalter in Jahren*	46,1 (± 21,1)	44,3 (± 21,4)	46,5 (± 20,6)	48,1 (± 21,3)	45,8 (± 21,1)

*±-Werte* Standardabweichung

Bei insgesamt 286 Patienten wurde ein Ethanolspiegel im Blut von mindestens 0,2 ‰ festgestellt, sodass 16,1 % aller eingelieferten Patienten alkoholintoxikiert waren. Hiervon waren 13,2 % der Patienten männlich und 2,8 % weiblich.

Als Marker des chronischen Alkoholkonsum bei nichtalkoholisierten, traumatologischen Schockraumpatienten wurden ein durchschnittliches mittleres, korpuskuläres Volumen (MCV) der Erythrozyten von 87,7 fl ± 4,7 fl (Normwert: 79–94 fl) und eine durchschnittliche Gammaglutamyltransferase(γ-GT)-Aktivität von 35,1 U/l ± 58,9 U/l (Normwert: 5–38 U/l) identifiziert. Bei alkoholisierten Patienten wurden ein durchschnittliches mittleres, korpuskuläres Volumen (MCV) der Erythrozyten von 90,4 fl ± 5,9 fl (Normwert: 79–94 fl) und eine durchschnittliche Gammaglutamyltransferase(γ-GT)-Aktivität von 75,1 U/l ± 120,5 U/l (Normwert: 5–38 U/l) als Marker des chronischen Alkoholkonsum erhoben (Tab. 2).

**Tab. 2:** Laborwerte der eingelieferten traumatologischen Schockraumpatienten

	Gesamtanzahl	2021	2022	2023	2024
*Akuter Marker*					
Ethanol ≥ 0,2 ‰	286	65	90	59	72
Prozentualer Anteil bezogen auf die Gesamtzahl	16,1 %	13,1 %	18,4 %	15,4 %	17,4 %
Weiblich	50 (2,8 %)	12 (2,4 %)	13 (2,7 %)	5 (1,3 %)	20 (4,8 %)
Männlich	236 (13,2 %)	53 (10,7 %)	77 (15,7 %)	54 (14,1 %)	52 (12,6 %)
Ethanolspitzenwert in ‰	5,06	2,29	3,86	3,90	5,06
*Chronischer Marker bei nichtalkoholisierten Patienten*					
MCV in fl	87,7 (± 4,7)	88,4 (± 4,1)	86,7 (± 4,2)	87,2 (± 4,7)	88,3 (± 4,2)
γ‑GT in U/I	35,1 (± 58,9)	34,6 (± 60,5)	34,5 (± 60,2)	35,5 (± 57,9)	35,9 (± 58,4)
*Chronischer Marker bei alkoholisierten Patienten*					
MCV in fl	90,4 (± 5,9)	89,4 (± 5,2)	89,9 (± 5,3)	90,8 (± 4,8)	91,4 (± 5,7)
γ‑GT in U/I	75,1 (± 120,5)	74,7 (± 117,5)	75,8 (± 118,7)	75,5 (± 124,7)	74,6 (± 121,8)

*±-Werte* Standardabweichung

Zeitlich wurden hierbei in den späten Abend- und frühen Morgenstunden sowie an den Wochenendtagen Samstag und Sonntag die meisten alkoholintoxikierten Patienten beobachtet. Die wenigsten Alkoholunfälle ereigneten sich montags. Im Verlauf der Woche kam es zu einer stetigen Zunahme alkoholisierter Verletzte, bevor der Höhepunkt am Samstag erreicht wurde.

**Abb. 1:**
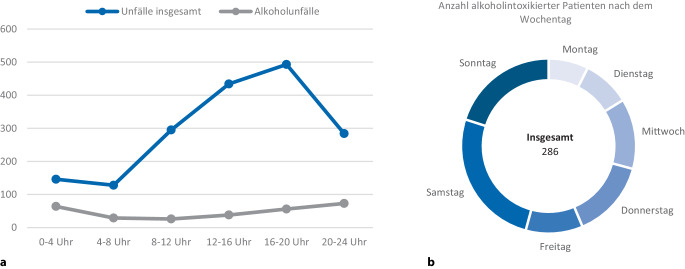
Alkoholunfälle, differenziert nach der Tages- (**a**) und Wochenzeit (**b**)

Uhrzeitlich verunfallten die meisten Patienten am Nachmittag zwischen 12 Uhr und 20 Uhr, wobei hierbei etwa jeder 10. Patient einen Blutalkoholspiegel ≥ 0,2 ‰ aufwies. Die wenigsten Alkoholunfälle ereigneten sich in den Morgenstunden zwischen 4 Uhr und 12 Uhr. In der Zeit zwischen 0 Uhr und 4 Uhr konnte bei 43,8 % der eingelieferten Schockraumpatienten Ethanol im Blut nachgewiesen werden (Abb. 1).

**Abb. 2:**
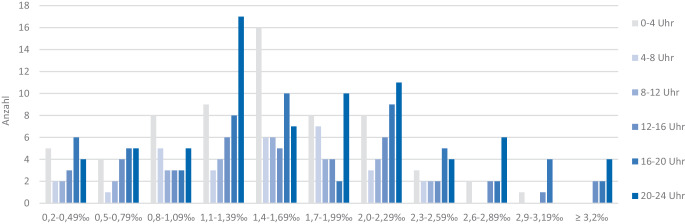
Blutalkoholspiegel in Promille nach der Tageszeit

Wird der Blutalkoholspiegel im Verhältnis zur Tageszeit korreliert, ist festzustellen, dass Patienten mit erhöhten Promillewerte > 2,6 ‰ ausschließlich ab den Mittagsstunden bis morgens um 4 Uhr verunfallen und als traumatologischer Schockraum eingeliefert werden. Die meisten alkoholintoxikierten Patienten werden in der Zeit zwischen 20 Uhr und 4 Uhr eingeliefert und haben einen Blutalkoholspiegel zwischen 1,1 ‰ und 1,69 ‰ (Abb. 2).

**Abb. 3:**
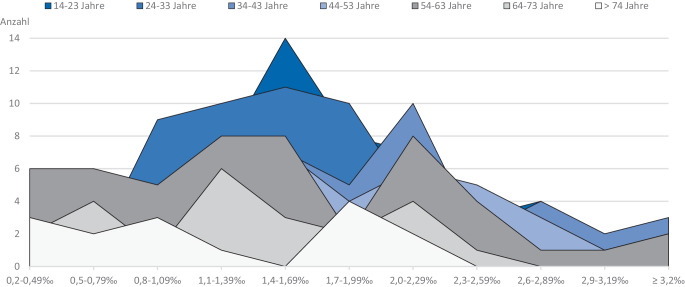
Korrelation des Blutalkoholpromillewerts mit dem Alter der verunfallten Patienten

Die kombinierte Betrachtungsweise nach der Höhe des Promillewerts und dem Alter verunfallter Patienten zeigt auf, dass besonders häufig in den Altersgruppen zwischen 34 und 43 Jahren, 44 und 53 Jahren sowie 54 und 63 Jahren erhöhte Blutpromillewerte > 2,3 ‰ detektiert werden konnten. Der gemessene Spitzenwert lag bei 5,06 ‰ im Blut nach unmittelbarer Einlieferung im Schockraum. Die meisten Patienten in der Altersgruppe zwischen 14 und 23 Jahren bzw. 24 und 33 Jahren wiesen einen Blutalkoholwert zwischen 0,8 ‰ und 2,3 ‰ auf. Vergleichbare Werte fanden sich auch bei Patienten ab 64 Jahren und älter (Abb. 3).

Bezüglich des Unfallmechanismus zeigten alle aufgenommenen Unfallpatienten etwa den gleichen Verletzungsgrad mit einem durchschnittlichen Injury Severity Score zwischen 9,4 und 14,4. Von 1104 Patienten, die nach einem Verkehrsunfall eingeliefert wurden, konnte bei 142 Patienten (12,9 %) Ethanol im Blut nachgewiesen werden. Hierbei zeigten sich Anteile von 11,9 % im Jahr 2021, 14,1 % im Jahr 2022, 9,6 % im Jahr 2023 und 18,8 % im Jahr 2024. Der durchschnittliche Blut-Promille-Gehalt lag bei 1,47 ‰ ± 0,81 ‰. Patienten, die nach einem Treppensturz in den traumatologischen Schockraum eingeliefert wurden, waren in 24,3 % der Fälle alkoholintoxikiert. Der durchschnittliche Blut-Promille-Gehalt lag hierbei bei 1,65 ‰ ± 0,83 ‰. Bei 19,2 % der Patienten konnte nach einem Sturz aus großer Höhe Ethanol nachgewiesen werden, wobei ein durchschnittlicher Ethanolgehalt von 1,47 ‰ ± 0,84 ‰ festgestellt wurde. Bei Patienten nach einem Stolpersturz konnte in 29,7 % der Patienten Ethanol nachgewiesen werden, sodass der durchschnittliche Alkoholgehalt bei 1,89 ‰ ± 0,82 ‰ lag. Lag eine Einklemmung als Unfallmechanismus vor, konnte bei 10,4 % der Patienten Alkohol im Blut nachgewiesen werden. Der durchschnittliche Blut-Promille-Gehalt lag bei 1,47 ‰ ± 0,50 ‰.

Insgesamt verstarben 23 von insgesamt 1780 eingelieferten Patienten im analysierten Zeitraum von 2021 bis 2024 im Schockraum an ihren schweren Verletzungen und 67 weitere Patienten innerhalb der nächsten 4 Wochen während ihres Krankenhausaufenthalts. Hiervon waren 17,6 % der Verunfallten alkoholisiert mit einem durchschnittlichen Ethanolgehalt von 1,23 ‰ ± 0,85 ‰ (Tab. 3).

**Tab. 3:** Unfallart, Verletzungsschwere und die Korrelation mit der Alkoholintoxikation

	Gesamtanzahl	2021	2022	2023	2024
*Verkehrsunfälle*	1097	312	288	204	233
Injury Severity Score (ISS)	11,4 ± 14,6	10,9 ± 15,3	10,7 ± 13,0	13,9 ± 16,4	12,3 ± 14,3
Exitus im Schockraum	16 (1,5 %)	7 (2,2 %)	0 (0 %)	3 (1,4 %)	6 (2,6 %)
Exitus innerhalb 4 Wochen	22 (2,1 %)	6 (1,9 %)	3 (1 %)	6 (2,9 %)	7 (3,0 %)
Ethanolgehalt bestimmt	945 (90,5 %)	291 (93,3 %)	259 (89,3 %)	184 (88,5 %)	211 (90,2 %)
Ethanol ≥ 0,2 ‰ im Blut	142 (12,9 %)	37 (11,9 %)	41 (14,1 %)	20 (9,6 %)	44 (18,8 %)
Durchschnittlicher Blut-Promille-Gehalt	1,47 ± 0,81	1,50 ± 0,68	1,54 ± 0,69	1,21 ± 0,69	1,48 ± 1,05
*Treppenstürze*	148	43	40	37	28
Injury-Severity-Score (ISS)	13,6 ± 14,9	15,8 ± 19,6	14,0 ± 11,8	12,4 ± 14,9	11,3 ± 9,5
Exitus im Schockraum	1 (0,7 %)	1 (2,3 %)	0 (0 %)	0 (0 %)	0 (0 %)
Exitus innerhalb 4 Wochen	12 (8,1 %)	3 (7,0 %)	3 (7,5 %)	3 (8,1 %)	3 (10,7 %)
Ethanol abgenommen	129 (87,2 %)	36 (83,7 %)	34 (85,0 %)	35 (94,6 %)	24 (85,7 %)
Ethanol ≥ 0,2 ‰ im Blut	36 (24,3 %)	7 (16,3 %)	13 (32,5 %)	10 (27 %)	6 (21,4 %)
Durchschnittlicher Blut-Promille-Gehalt	1,65 ± 0,83	1,37 ± 0,64	1,63 ± 0,90	1,99 ± 0,87	1,42 ± 0,78
*Sturz großer Höhe*	144	28	45	19	52
Injury Severity Score (ISS)	14,4 ± 15,8	10,38 ± 15,4	17,6 ± 15,6	14,5 ± 18,6	13,8 ± 15,0
Exitus im Schockraum	3 (2,1 %)	1 (3,4 %)	1 (2,2 %)	0 (0 %)	1 (1,9 %)
Exitus innerhalb 4 Wochen	6 (4,1 %)	0 (0 %)	3 (6,7 %)	2 (10,5 %)	1 (1,9 %)
Ethanol abgenommen	129 (88,4 %)	28 (96,6 %)	40 (88,9 %)	17 (89,5 %)	44 (83,0 %)
Ethanol ≥ 0,2 ‰ im Blut	28 (19,2 %)	7 (24,1 %)	6 (13,3 %)	7 (36,8 %)	8 (15,1 %)
Durchschnittlicher Blut-Promille-Gehalt	1,47 ± 0,84	1,18 ± 0,79	2,13 ± 0,78	1,58 ± 1,00	1,25 ± 0,68
*Stolpersturz*	138	31	39	35	33
Injury Severity Score (ISS)	10,1 ± 9,9	13,1 ± 8,9	9,3 ± 10,8	8,5 ± 10,5	10,2 ± 8,9
Exitus im Schockraum	2 (1,4 %)	0 (0 %)	1 (2,6 %)	0 (0 %)	1 (3,0 %)
Exitus innerhalb 4 Wochen	15 (10,9 %)	5 (16,1 %)	4 (10,3 %)	6 (17,1 %)	0 (0 %)
Ethanol abgenommen	123 (89,1 %)	28 (90,3 %)	38 (97,6 %)	28 (80 %)	29 (87,9 %)
Ethanol ≥ 0,2 ‰ im Blut	41 (29,7 %)	4 (12,9 %)	17 (43,6 %)	13 (37,1 %)	7 (21,2 %)
Durchschnittlicher Blut-Promille-Gehalt	1,89 ± 0,82	1,96 ± 0,31	2,01 ± 1,00	2,01 ± 0,65	1,40 ± 0,67
*Leiterstürze*	89	36	17	25	11
Injury Severity Score (ISS)	13,8 ± 12,7	16,1 ± 12,6	17,1 ± 14,6	11,6 ± 12,3	6,8 ± 6,7
Exitus im Schockraum	0	0	0	0	0
Exitus innerhalb 4 Wochen	1 (1,1 %)	0 (0 %)	1 (5,9 %)	0 (0 %)	0 (0 %)
Ethanol abgenommen	83 (93,3 %)	36 (100 %)	16 (94,1 %)	22 (88 %)	11 (100 %)
Ethanol ≥ 0,2 ‰ im Blut	6 (4,5 %)	2 (5,6 %)	0 (0 %)	3 (12 %)	0 (0 %)
Durchschnittlicher Blut-Promille-Gehalt	1,11 ± 0,62	1,25 ± 0,21	0,0 ± 0,0	1,40 ± 0,60	0,0 ± 0,00
*Einklemmung*	67	7	14	25	21
Injury Severity Score (ISS)	9,4 ± 10,4	6,9 ± 6,6	7,3 ± 8,4	12,6 ± 12,6	7,7 ± 9,1
Exitus im Schockraum	0 (0 %)	0 (0 %)	0 (0 %)	0 (0 %)	0 (0 %)
Exitus innerhalb 4 Wochen	1 (1,5 %)	0 (0 %)	0 (0 %)	0 (0 %)	1 (4,8 %)
Ethanol abgenommen	59 (88,1 %)	7 (100 %)	11 (78,6 %)	23 (92,0 %)	18 (85,7 %)
Ethanol ≥ 0,2 ‰ im Blut	7 (10,4 %)	0 (0 %)	1 (7,1 %)	2 (8,0 %)	4 (19 %)
Durchschnittlicher Blut-Promille-Gehalt	1,47 ± 0,50	0,0 ± 0,00	2,08 ± 0,00	0,86 ± 0,06	1,62 ± 0,28
*Verstorbene Patienten*					
Gesamtzahl	90	24	20	24	22
Alkoholisierte Verstorbene	16	6	3	3	4
Durchschnittlicher Blut-Promille-Gehalt	1,23 ± 0,85	1,51 ± 1,10	0,92 ± 0,69	1,02 ± 0,95	1,26 ± 0,71

*±-Werte* Standardabweichung

## Diskussion

International ist Alkohol einer der führenden Risikofaktoren für Krankenhausaufenthalte, Verletzungen und Unfälle. Im Jahr 2021 starben etwa 1,8 Mio. Menschen weltweit an den Folgen ihres Alkoholkonsums. Ebenso ist laut den Ergebnissen der Global-Burden-of-Disease-Studie Alkoholgenuss auf Platz 10 einer der Hauptrisikofaktoren für vorzeitige Sterblichkeit und verlorene Lebensjahre infolge von Krankheit und Behinderung [11]. So entstehen in Deutschland allein jedes Jahr volkswirtschaftliche Kosten von ca. 57 Mrd. € durch Alkoholkonsum und dessen Folgen. Diese Aufwendungen umfassen sowohl direkte medizinische Ausgaben als auch indirekte Kosten durch Produktivitätsverluste, vorzeitige Verrentung und Sterblichkeit [12].

Gmell et al. kamen 2007 bei ihrer Studie aus der Schweiz zu dem Fazit, dass alkoholbedingte Verletzungen nicht nur ein Problem einer kleinen, stark konsumierenden Minderheit sind, sondern große Bevölkerungsteile betreffen [9]. Auch in der vorliegenden Studie zeigte sich, dass 16,1 % aller traumatologischen Schockraumpatienten Alkohol konsumiert haben. So war ein weiteres Fazit, dass selbst kleine Mengen von Ethanol mit erhöhten Risiken für Verletzungen assoziiert sind und präventive Maßnahmen an der Allgemeinbevölkerung ausgerichtet sein müssten [9].

Laut Statistischem Bundesamt haben 70,4 % der Pkw-Fahrer, die unter Alkoholeinfluss einen Unfall mit Personenschaden im Jahr 2021 verursacht haben, einen Blut-Promille-Wert von mindestens 1,1 ‰ [8]. Auch zeigt unsere Studie, dass die meisten, verunfallten Patienten, die alkoholintoxikiert in den Schockraum eingeliefert wurden, einen Promillewert von 1,55 ‰ ± 0,80 ‰ hatten. Patienten, die an ihren schweren Verletzungen verstarben und alkoholintoxikiert eingeliefert wurden, wiesen im Durchschnitt ein Blutalkohol-Promille-Wert von 1,23 ‰ ± 0,85 ‰ auf.

Als präventive Maßnahme zur Senkung alkoholbedingter Unfälle im Straßenverkehr wurde vom Bundesgerichtshof, historisch gesehen, erstmals 1953 eine Promillegrenze von 1,5 ‰ für Autofahrer eingeführt. Diese wurde im Jahr 1966 auf 1,3 ‰ hinabgestuft. Ab dem 26.07.1973 galt eine Promillegrenze von 0,8 ‰, und erstmals wurde festgesetzt, dass bei Überschreitung eine Ordnungswidrigkeit gemäß § 24a des Straßenverkehrsgesetzes entstand. Am 01.05.1998 wurde diese Grenze auf 0,5 ‰ reduziert [13]. Die Polizei konnte und kann jedoch ab einem Alkoholwert von 0,15 mg/l Alkohol in der Atemluft, entsprechend 0,3 ‰, bei im Straßenverkehr auffällig gewordene Untersuchten einen Unfall als alkoholbeeinflusst einstufen [8].

Durch die präventive Einführung der Promillegrenze wird seitdem ein kontinuierlicher Rückgang alkoholbedingter Verkehrsunfälle verzeichnet [13].

Zudem wird seit 2012 als weitere vorbeugende Maßnahme in Deutschland mit führenden Unfallkliniken ein bundesweites Unfallpräventionsprogramm der Deutschen Gesellschaft für Unfallchirurgie durchgeführt. P.A.R.T.Y. ist die Abkürzung für „Prevent Alcohol and Risk-Related Trauma in Youth“ und stammt aus Kanada. Frei übersetzt geht es um die Prävention von durch Alkohol und risikoreiches Verhalten verursachte Verletzungen bei Jugendlichen. Hierbei wird durch eine Hospitation in der Notaufnahme und auf der Intensivstation im Krankenhaus über Verletzungen, die durch risikoreiches Verhalten im Straßenverkehr verursacht werden, aufgeklärt [14].

Doch trotz dieser präventiven Maßnahmen ist seit 2021, nach jahrelanger Abnahme, ein Wiederanstieg alkoholbedingter Unfälle im Straßenverkehr zu detektieren. Parallel dazu nimmt die Zahl der alkoholisierten Patienten im traumatologischen Schockraum zu. In unserer Analyse zeigten sich Anteile von 13,1 % im Jahr 2021, 18,4 % im Jahr 2022, 15,4 % im Jahr 2023 und 17,4 % im Jahr 2024. Mögliche Gründe hierfür bleiben unklar.

## Limitationen

Obwohl die Ergebnisse dieser Untersuchung vielversprechend sind, gibt es zu berücksichtigende Limitationen. Erstens beschränkt sich die Analyse auf das Universitätsklinikum Regensburg in Bayern, weshalb eine Generalisierung der Ergebnisse nur eingeschränkt möglich ist. Zukünftige Ergebnisse sollten daher eine größere Stichprobe miteinbeziehen, um eine größere Übertragbarkeit der Ergebnisse zu erreichen. Zweitens ist aufgrund des retrospektiven Studiendesigns kein Kausalzusammenhang zwischen der Verletzungsschwere und dem Alkoholpromillegehalt möglich. Es wurde der Ethanolgehalt bei 89,8 % aller Schockraumpatienten erhoben.

## Ausblick

Zusammenfassend lässt sich festhalten, dass Alkohol auch heute noch ein entscheidender Risikofaktor für Unfälle im Straßenverkehr, Verletzungen und Sterblichkeit darstellt. Die vorliegende Studie liefert wichtige Daten zum wahren Ausmaß alkoholbedingter Unfälle in der Schwerverletztenversorgung.

Das Bewusstsein für den Risikofaktor Alkohol muss geschärft und erneut in den Fokus gestellt werden. Denn nur durch zusätzliche volksnahe, präventive Maßnahmen, wie Aufklärungen, aber auch ggf. Verbote und Sanktionen, kann eine weitere Abnahme risikoreichen Alkoholkonsums in der Bevölkerung erreicht werden.

## Fazit für die Praxis

- Bereits seit Langem ist bekannt, dass es einen kausalen Zusammenhang zwischen dem Alkoholkonsum einer Bevölkerung und der Häufigkeit von Unfällen oder Verletzungen gibt.
- Alkohol ist auch heute noch ein entscheidender Risikofaktor für Unfälle im Straßenverkehr, Verletzungen und Sterblichkeit.
- Patienten, die alkoholisiert im traumatologischen Schockraum eines universitären Maximalversorgers eingeliefert wurden, wiesen im Durchschnitt einen Promillewert von 1,55 ± 0,80 ‰ auf.
- In der Zeit zwischen 0 Uhr und 4 Uhr konnte bei 43,8 % der eingelieferten Schockraumpatienten Ethanol im Blut nachgewiesen werden.
- Trotz präventiver Maßnahmen muss das Bewusstsein für den Risikofaktor Alkohol wieder geschärft und in den Fokus gestellt werden.

## Data Availability

Daten auf Anfrage verfügbar.

## References

[1] Cherpitel CJ (2007) Alcohol and injuries: a review of international emergency room studies since 1995. Drug Alcohol Rev 26(2):201–214. 10.1080/09595230601146686 (PMID: 17364856) 17364856 10.1080/09595230601146686

[2] Cherpitel CJ (2013) Focus on: the burden of alcohol use—trauma and emergency outcomes. Alcohol Res 35(2):150–154 (PMID: 24881323, PMCID: PMC3908706) 24881323 10.35946/arcr.v35.2.05 PMC3908706

[3] Rehm J, Gmel GE Sr, Gmel G, Hasan OSM, Imtiaz S, Popova S, Probst C, Roerecke M, Room R, Samokhvalov AV, Shield KD, Shuper PA (2017) The relationship between different dimensions of alcohol use and the burden of disease—an update. Addiction 112(6):968–1001. 10.1111/add.13757 (Epub 2017 Feb 20, PMID: 28220587, PMCID: PMC5434904) 28220587 10.1111/add.13757 PMC5434904

[4] World Health Organization (2018) Global status report on alcohol and health

[5] Manthey J, Shield KD, Rylett M, Hasan OSM, Probst C, Rehm J (2019) Global alcohol exposure between 1990 and 2017 and forecasts until 2030: a modelling study. Lancet 393(10190):2493–2502. 10.1016/S0140-6736(18)32744-2 (PMID: 31076174) 31076174 10.1016/S0140-6736(18)32744-2

[6] World Health Organization (2024) Global status report on alcohol and health and treatment of substance use disorders

[7] Bundesministerium für Gesundheit (2025) Sucht und Drogen. https://www.bundesgesundheitsministerium.de/themen/praevention/gesundheitsgefahren/sucht-und-drogen.html. Zugegriffen: 10. Jan. 2025

[8] Statistisches Bundesamt (2021) Verkehrsunfälle. Unfälle unter dem Einfluss von Alkohol oder anderen berauschenden Mitteln im Straßenverkehr 2021

[9] Gmel G, Kuendig H, Kuntsche S, Daeppen JB (2007) Alkohol und Verletzungen: Alkoholkonsum, bezogene Risiken und attributive Anteile. Eine Studie in der Notfallaufnahme der Lausanner Universitätsklinik (CHUV)

[10] Jastrzębska I, Zwolak A, Szczyrek M, Wawryniuk A, Skrzydło-Radomańska B, Daniluk J (2016) Biomarkers of alcohol misuse: recent advances and future prospects. Prz Gastroenterol 11(2):78–89. 10.5114/pg.2016.60252 (PMID: 27350834, PMCID: PMC4916243) 27350834 10.5114/pg.2016.60252 PMC4916243

[11] Institute for Health Métrics and Evaluation. Global burden of disease. Zugegriffen: 10. Jan. 2025

[12] Bundesministerium für Gesundheit. Alkoholkonsum in Deutschland: Zahlen und Fakten. Zugegriffen: 10. Jan. 2025

[13] Bußgeldkatalog. Promillegrenze in Deutschland – Alkohol am Steuer 2024. Zugegriffen: 10. Jan. 2025

[14] Deutsche Gesellschaft für Unfallchirurgie e.V. (DGU) (2025) P.A.R.T.Y. – Unfallprävention für Jugendliche. https://www.dgu-online.de/versorgung-wissenschaft/qualitaet-und-sicherheit/party-unfallpraevention-fuer-jugendliche. Zugegriffen: 10. Jan. 2025

